# Management of Myasthenic Crisis and Emerging Roles of Molecularly Targeted Therapies: A Narrative Review

**DOI:** 10.3390/neurolint17100163

**Published:** 2025-10-08

**Authors:** Seiya Takahashi, Ryuta Kinno

**Affiliations:** Department of Neurology, Showa Medical University Fujigaoka Hospital, 1-30 Fujigaoka Aoba-Ku, Yokohama-Shi 227-8501, Kanagawa, Japan

**Keywords:** myasthenia gravis, myasthenic crisis, FcRn inhibitors, complement inhibitors

## Abstract

Myasthenia gravis (MG) is a chronic autoimmune disorder characterized by fluctuating skeletal muscle weakness. Myasthenic crisis (MCr), a severe and potentially life-threatening complication, presents with respiratory failure and requires intensive care and rapid immunomodulatory intervention. Conventional MCr treatments—such as plasma exchange (PLEX), intravenous immunoglobulin (IVIG), and intravenous methylprednisolone (IVMP)—remain standard treatments; however, they present significant limitations, including delayed onset of action, adverse effects, and inconsistent efficacy. Recent therapeutic advances have led to the development of molecularly targeted therapies based on MG pathophysiology, particularly neonatal Fc receptor (FcRn) inhibitors and complement inhibitors, which have shown efficacy in refractory or maintenance settings. This review explores the potential application of these agents in MCr. We review published case reports involving FcRn inhibitors (efgartigimod, efgartigimod-SC, rozanolixizumab) and complement inhibitors (eculizumab, ravulizumab, zilucoplan), highlighting their rapid onset of action and safety profiles in MCr. While efgartigimod and eculizumab are the most commonly reported agents in MCr, data remain limited to small case series. Emerging evidence suggests these agents may offer effective alternatives to conventional therapies, with favorable safety and potential for rapid symptom resolution. We also discuss strategic considerations for therapy selection, including antibody subtype, coexisting autoimmune conditions, genetic factors, and transition to long-term maintenance. Though the current evidence is promising, large-scale randomized studies are needed to establish definitive roles for these therapies in MCr management.

## 1. Introduction

Myasthenia gravis (MG) is an autoimmune neuromuscular disorder caused by immune-mediated disruption at the neuromuscular junction (NMJ), which is characterized by progressive and fatigable muscle weakness. While the majority of patients possess autoantibodies against the acetylcholine receptor (AChR), antibodies targeting muscle-specific kinase (MuSK) and low-density lipoprotein receptor-related protein 4 (LRP4) have also been identified, indicating that MG comprises diverse subtypes based on antibody profiles [1].

MG has a prevalence of about 15–25 cases per 100,000 in Western countries [2] and 23.1 per 100,000 in Japan [3]. It shows female onset in the 20s–30s and male onset after 50. MG is classified by age of onset, antibody profile, clinical type, and thymic pathology. Early-onset MG is often linked to thymic hyperplasia, while the late-onset type affects older men with fewer thymic abnormalities [4]. About 80–85% of patients have anti-AChR antibodies and 5–8% anti-MuSK antibodies, the latter associated with severe bulbar/respiratory involvement and steroid resistance [5]. Other antibodies (anti-LRP4, anti-agrin) have been reported, mostly in mild ocular MG [6]. Clinically, MG is divided into ocular and generalized forms, with myasthenic crisis requiring urgent care. Thymic pathology includes hyperplasia or thymoma (10–15% of cases), the latter being linked to severe disease and autoimmune complications [7]. Prognosis varies by subtype.

Clinically, MG manifests a broad spectrum of disease severity, ranging from mild ocular myasthenia limited to the extraocular muscles to generalized forms involving respiratory muscle paralysis. Among them, myasthenic crisis (MCr)—defined by respiratory failure—is a life-threatening emergency that demands prompt and targeted therapeutic intervention [2].

Conventionally, treatment for MG centers on immunomodulatory therapy with corticosteroids and immunosuppressants. In MCr, plasma exchange (PLEX), intravenous immunoglobulin therapy (IVIG), and high-dose intravenous methylprednisolone (IVMP) have been used; however, these treatments pose challenges including variability in individual treatment responses, adverse events, and treatment resistance [8,9,10].

Against this background, recent advances have been made in developing novel therapeutics based on the molecular pathogenesis of MG, with particular attention to molecularly targeted therapies such as neonatal Fc receptor (FcRn) inhibitors and complement inhibitors. Their clinical efficacy as maintenance therapies for refractory MG has been increasingly demonstrated in clinical trials [11]. However, evidence regarding their efficacy in MCr remains limited to a small number of case reports. This article summarizes the current status and challenges of MCr treatment and provides a comprehensive review of the potential and future perspectives of molecularly targeted therapies applied in MCr, based on the latest clinical findings and case reports.

## 2. Conventional Treatments for MG, Including Myasthenic Crisis

### 2.1. Immunopathological Mechanisms of Myasthenia Gravis

**Figure 1:**
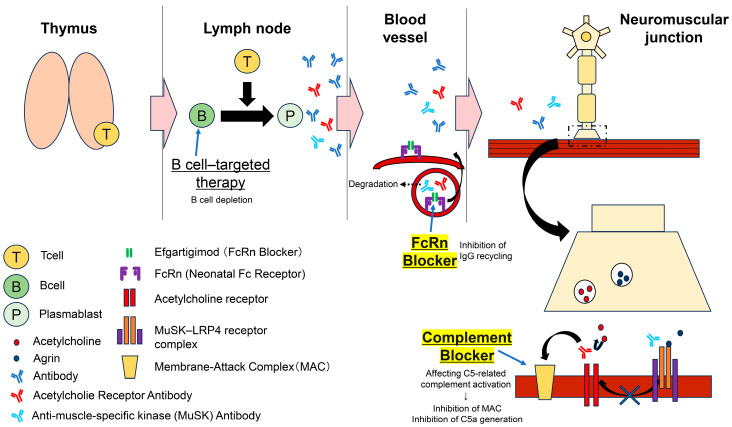
Immunopathogenesis of myasthenia gravis and mechanisms of action of emerging molecularly targeted therapies.

Before discussing therapeutic strategies for MG, this article provides an overview of the immunological background of the disease. A simplified diagram of the immunopathological mechanisms of MG is presented in Figure 1, and the details of these mechanisms are described below. In AChR antibody-positive MG, thymic abnormalities are considered the initiating factor of the disease. Immunological changes in thymoma associated with MG, such as the formation of germinal centers and an increase in follicular helper T cells, have been reported [12,13]. It is also well established that mature B cells in the thymus serve as a source of autoantibody production [14]. Self-reactive B cells activated in the thymus migrate to the bone marrow or secondary lymphoid tissues, where they receive stimulation from activated CD4+ helper T cells—particularly T follicular helper cells—and differentiate into long-lived autoreactive plasma cells that continuously produce pathogenic autoantibodies [15]. These pathogenic autoantibodies enter the bloodstream via lymphatic vessels and are distributed systemically. The homeostasis of these IgG autoantibodies critically depends on FcRn. FcRn is widely expressed in various tissues and cells, including epithelial cells, vascular endothelial cells, hematopoietic cells, intestinal epithelium, kidney, liver, and placenta, and it plays an essential role in IgG recycling and transcytosis [16]. FcRn binds to the Fc region of IgG, specifically at the interface between the CH2 and CH3 domains. IgG is first nonspecifically internalized into cells where, under the acidic environment of the endosome (pH ~6.0), it binds FcRn with high affinity. This binding protects IgG from lysosomal degradation, after which the complex is transported to the cell membrane and released back into circulation at neutral pH [17]. This recycling mechanism extends the serum half-life of IgG to approximately 21 days, allowing pathogenic autoantibodies to circulate for a prolonged period. This persistent circulation contributes to the chronicity and relapse of symptoms. When these circulating autoantibodies reach the NMJ, they induce pathophysiological changes through mechanisms that vary according to the antibody subtype. The majority of MG patients harbor autoantibodies targeting the AChR on the postsynaptic membrane at the NMJ. These antibodies block acetylcholine binding, promote internalization of the AChR, and trigger complement-mediated tissue injury, ultimately impairing neuromuscular transmission. These autoantibodies are predominantly of the IgG1 and IgG3 subclasses, which possess potent classical complement pathway-activating properties [18]. Upon binding to AChR, the Fc region of the antibody recruits the C1 complex (C1q, C1r, C1s), initiating activation of the classical complement cascade. The complement system plays a critical role in both innate and adaptive immunity, and its dysregulation can compromise host defense and inflammatory responses, contributing to infections and autoimmune diseases [19]. Specifically, activation of the C1 complex leads to cleavage of C4 and C2, forming the C4b2a complex (C3 convertase). C3 convertase cleaves C3 into C3a and C3b, with C3b contributing to the formation of C5 convertase. Subsequent cleavage of C5 generates C5a and C5b; C5b sequentially binds to C6, C7, C8, and C9 to form the membrane attack complex (MAC) [20]. The MAC creates pores in the muscle cell membrane, altering its permeability, causing structural damage, and impairing synaptic transmission—one of the central pathophysiological mechanisms of MG [21]. Recent studies have reported significantly elevated serum levels of the complement activation markers C3a, C5a, and sC5b-9 in patients with AChR antibody-positive MG, supporting the involvement of complement cascade activation in MG pathogenesis [22].

In contrast, MuSK antibody-positive MG shows minimal association with thymic pathology, and the autoantibodies are produced primarily by plasma cells. In MuSK antibody-positive patients, the Th2 cytokine IL-10 is considered a key factor promoting class switching to IgG4. Additionally, the HLA-DRB1*14 allele has been detected at a high frequency, suggesting a genetic background associated with IgG4 class switching [23]. The produced anti-MuSK antibodies circulate in the bloodstream and reach the NMJ, where they interfere with the interaction between MuSK and LRP4, disrupting downstream signaling. Consequently, agrin-dependent clustering of AChRs is impaired, leading to insufficient accumulation and stabilization of AChRs and resulting in structural and functional disruption of the NMJ. These anti-MuSK antibodies are predominantly of the IgG4 subclass, which has limited complement activation capacity, explaining the absence of complement-mediated tissue injury in this subtype [14].

Although this study focuses on MCr, the molecular mechanisms underlying it remain incompletely understood. It has been reported that patients with MCr often have higher mean levels of anti-AChR antibodies than those with milder disease [24], and marked increases in antibody titers have been linked to the onset of MCr [25]. In a previous study of intercostal muscle samples from MCr patients, extensive destruction of the postsynaptic membrane at the NMJ was observed, suggesting that complement activation plays a crucial role in NMJ pathology in this setting [26]. Additionally, selective activation of specific inflammatory and regulatory cytokines has been reported in MCr [27]. These findings provide important insights into the mechanisms of disease exacerbation and the identification of therapeutic targets. In summary, the immunopathology of MG varies by antibody subtype and, in MCr, antibody titers and complement activation appear to be key contributors. Based on these insights, the following section provides an overview of the major therapeutic targets for MG.

T cells mature in the thymus and assist B cells in differentiating into plasma cells within the lymph nodes. Plasma cells produce various antibodies, including anti-AChR and anti-MuSK antibodies. These antibodies circulate in the bloodstream and bind FcRn, which protects them from degradation. The antibodies then migrate to the neuromuscular junction, where they bind to receptors and inhibit signal transduction, thereby suppressing muscle contraction. B cell-targeted therapies reduce the number of B cells, while FcRn inhibitors block the binding between FcRn and antibodies, disrupting antibody recycling and promoting antibody degradation. Complement inhibitors mainly suppress the activation of C5, thereby inhibiting the complement cascade.

### 2.2. Therapeutic Targets for MG

The treatment of MG aims to control autoimmune responses at the NMJ and improve neuromuscular transmission. Broadly, therapies can be divided into non-specific immunosuppressive therapies and molecularly targeted therapies.

Conventional immunosuppressive therapies are non-specific. In MCr, PLEX, IVIG, and IVMP are commonly used while, in the maintenance phase, corticosteroids, calcineurin inhibitors such as tacrolimus and cyclosporine, and other immunosuppressants are employed. The targets of these drugs include corticosteroids (e.g., prednisolone), which primarily suppress T cells and reduce inflammatory cytokine production through the glucocorticoid receptor-mediated inhibition of NF-κB and AP-1 transcription [28]. Tacrolimus and cyclosporine inhibit calcineurin, thereby suppressing IL-2 transcription, which is essential for T-cell activation and blocking T-cell differentiation and proliferation [29]. As a treatment for MCr, PLEX physically removes pathogenic IgG and complement components, while IVIG exerts multifaceted immunomodulatory effects, including FcRn competitive inhibition, complement pathway suppression, and modulation of autoreactive lymphocyte function [30]. Although the precise mechanism of IVMP remains unclear, proposed effects include increased acetylcholine availability [31], suppression of complement-mediated injury [32], and inhibition of T cells and cytokines [33].

In recent years, molecularly targeted therapies such as complement inhibitors and FcRn inhibitors have been introduced clinically. Complement inhibitors block C5 cleavage at the terminal stage of the complement cascade, preventing formation of the MAC and thereby protecting the postsynaptic membrane from destruction in AChR antibody-positive MG. Proximal complement functions, including C3 activation, C3b-mediated opsonization, and immune complex clearance, are preserved [34,35] (Figure 1). FcRn inhibitors bind FcRn with high affinity, inhibiting IgG recycling and selectively, reversibly lowering the serum concentration of all IgG, including pathogenic autoantibodies. FcRn normally protects IgG from lysosomal degradation, maintaining serum IgG homeostasis; blockade of this mechanism by FcRn inhibitors reduces circulating IgG, including anti-AChR and anti-MuSK antibodies [36]. This effect is similar to that of PLEX in directly reducing antibodies but, unlike PLEX—which non-selectively removes IgA, IgM, and coagulation factors—FcRn inhibitors act specifically on IgG, offering safety advantages with a lower risk of infection and systemic immunosuppression.

In MCr, although the exact pathophysiological mechanisms remain unclear, antibody- and complement-mediated pathology appears to be involved, suggesting that these novel molecularly targeted therapies may represent promising treatment options.

### 2.3. Comparison of FcRn Inhibitors and Complement Inhibitors

Both FcRn inhibitors and complement inhibitors, as molecularly targeted therapies for MG, improve symptoms by controlling NMJ damage mediated by autoantibodies and the complement pathway. However, they differ markedly in mechanisms of action, pharmacokinetics, and clinical characteristics. FcRn inhibitors bind FcRn and inhibit IgG recycling, selectively and reversibly reducing the serum levels of all IgG, including pathogenic autoantibodies [36]. Complement inhibitors prevent C5 cleavage, suppress MAC formation, and thereby protect the postsynaptic NMJ from AChR antibody-dependent damage [34].

Regarding indications, FcRn inhibitors have a broad pharmacological spectrum, acting in anti-AChR antibody-positive, anti-MuSK antibody-positive, and even seronegative patients. They have also shown efficacy in MG patients with comorbid IgG-mediated autoimmune diseases, such as Stiff Person Syndrome [37] and GQ1b antibody syndrome [38]. In contrast, complement inhibitors are limited to anti-AChR antibody-positive MG, where complement-mediated pathology predominates.

There are differences in the onset of clinical effect. FcRn inhibitors reduce IgG levels within days, with clinical improvement observed in most cases within one to two weeks. Complement inhibitors typically show clinical effects within about one week, although some cases demonstrate significant symptom relief within days, making them effective for rapid control during MCr [39,40,41,42].

In terms of safety, FcRn inhibitors theoretically pose an infection risk due to IgG reduction; however, current reports indicate a low incidence of severe infections [36]. Complement inhibitors carry a significant risk of meningococcal infection, with reports in Japan indicating an incidence up to 6100 times higher in eculizumab-treated patients [43]. Therefore, meningococcal vaccination prior to treatment and prophylactic antibiotics as needed are recommended. If fever or altered consciousness occurs during complement inhibitor therapy, blood cultures should be obtained immediately under contact precautions, and intravenous third-generation cephalosporins should be started without delay, as delay in antimicrobial therapy for invasive meningococcal infections can be fatal within 24 h [44].

Economic considerations also differ. Both therapies are expensive, but long-term continuous administration of complement inhibitors poses a high financial burden. FcRn inhibitors allow for more flexible dosing regimens, enabling cost adjustments based on symptom control.

In emergency situations, such as in intensive care units, complement inhibitors can rapidly improve symptoms. FcRn inhibitors can be administered intravenously in MCr; however, their onset of effect is relatively slow, which may pose challenges for treatment strategies. At the same time, the risk of meningococcal infection during complement inhibitor therapy cannot be ignored, necessitating vaccination and, if required, concurrent prophylactic antibiotics.

Taken together, complement inhibitors are effective for sustained symptom control in anti-AChR antibody-positive MG and as rescue therapy during MCr, but infection risk and cost are concerns. FcRn inhibitors can be applied across a broad range of MG subtypes via reversible IgG reduction, offering flexibility in dosing, but their immediate efficacy in MCr is relatively limited. Therefore, optimizing MG treatment requires a comprehensive consideration of pharmacological characteristics, clinical efficacy, safety profiles, and cost-effectiveness to develop individualized therapeutic strategies based on patient background and disease status.

### 2.4. Conventional Treatments and Limitations in Myasthenic Crisis

MCr is a life-threatening condition characterized by bulbar and respiratory muscle paralysis, necessitating ventilatory support, and is experienced by 15–20% of MG patients during their lifetime [2]. Management includes intensive-care-level respiratory support, enteral nutrition, circulatory and infection control, and immunomodulatory therapies such as PLEX, IVIG, and IVMP.

PLEX physically removes pathogenic autoantibodies, including anti-AChR and anti-MuSK antibodies, providing rapid symptomatic relief [45]. However, it carries risks such as invasive vascular access, coagulopathy, hypoproteinemia, infection, and hemodynamic instability, and it requires specialized equipment and personnel. Moreover, PLEX may trigger “antibody overshoot,” a rebound increase in antibody levels that can cause symptom recurrence [25].

IVIG acts via multiple immunomodulatory mechanisms, including competitive binding to FcRn and inhibition of complement activation. Several studies have shown no significant difference in efficacy between IVIG and PLEX [17]. IVIG is particularly useful in patients with unstable hemodynamics or contraindications to PLEX, but its delayed onset is a limitation [45]. Additionally, issues such as supply shortages, renal impairment, thrombosis, and aseptic meningitis may arise. Responsiveness to IVIG is influenced by FcRn genetic polymorphisms, particularly variable number tandem repeats (VNTRs) in the FCGRT gene promoter region [46]. Patients with the VNTR3 allele exhibit higher FcRn expression and more efficient IgG recycling, resulting in better IVIG response, whereas VNTR2 carriers tend to have lower FcRn expression and a reduced response to IVIG.

IVMP is frequently used in MCr but may transiently worsen muscle weakness early in treatment. Administering IVMP immediately after IVIG or PLEX may mitigate this worsening [47]. While effective in ocular MG, clinicians should monitor for systemic side effects such as hyperglycemia, peptic ulcers, infection, psychiatric symptoms, electrolyte disturbances, and cardiovascular complications.

Although direct comparisons via RCTs are limited, both PLEX and IVIG have demonstrated efficacy [4,5]. In practice, treatment selection is guided by the patient’s overall condition and institutional resources. Since no significant difference in efficacy exists between PLEX and IVIG, the importance of individualized therapy is emphasized [3].

Despite advances in intensive care, MCr continues to be associated with high morbidity and mortality, and conventional therapies remain constrained. There is a growing need for more effective treatments, and the application of pathophysiology-based molecularly targeted therapies is gaining attention.

Currently available molecularly targeted therapies include FcRn inhibitors and complement inhibitors, both approved for maintenance therapy in refractory MG. However, evidence supporting their efficacy in MCr is limited to case reports and retrospective analyses, with no high-level clinical data from RCTs. Moreover, major trials—such as efgartigimod (EFG, ADAPT), subcutaneous efgartigimod (EFG-SC, ADAPT-SC), eculizumab (ECU, REGAIN), ravulizumab (RAV, CHAMPION MG), zilucoplan (ZIL, RAISE), and rozanolixizumab (ROZ, MycarinG)—all excluded MCr patients [11,34,48,49,50,51]. Nevertheless, these trials demonstrated improvements in the Myasthenia Gravis—Activities of Daily Living (MG-ADL) and Quantitative Myasthenia Gravis (QMG) scores within one to two weeks, theoretically supporting their potential use in MCr. Small-scale case reports comparing IVIG and EFG in patients at imminent risk of MCr have shown improvements in MG-ADL scores at weeks 1 and 4, suggesting that molecularly targeted therapies may complement—or potentially replace—conventional treatments in the future [52].

## 3. Novel Options for MCr Treatments: Current Status and Perspectives of Molecularly Targeted Therapies in MCr

### 3.1. FcRn Inhibitors and MCr

Among novel molecularly targeted therapies, FcRn inhibitors have emerged as a promising option for the treatment of MCr. In this section, we discuss the potential application of molecularly targeted therapy in MCr based on reported cases of FcRn inhibitor use.

Currently, three FcRn inhibitors—EFG, EFG-SC, and ROZ—are used clinically. EFG-SC is a subcutaneous formulation of EFG that contains hyaluronidase, enhancing tissue permeability and allowing for short-duration administration with efficacy comparable to the intravenous formulation, thereby improving dosing convenience [48]. All FcRn inhibitors are administered on a weekly schedule, although the dosing method and number of doses per cycle differ: EFG is administered intravenously four times per cycle, EFG-SC subcutaneously four times per cycle, and ROZ subcutaneously six times per cycle. Typically, subsequent cycles are initiated before symptom relapse, with intervals adjusted according to patient status. Structurally, EFG is an Fc fragment of human IgG1 with a relatively small molecular weight, whereas ROZ is a full-length humanized anti-FcRn monoclonal antibody with a larger molecular weight. ROZ exhibits high FcRn affinity not only under acidic intracellular conditions, but also at neutral pH on the cell surface [53]. However, the clinical implications of these structural and pharmacological differences remain unclear.

We conducted a search of PubMed and Google Scholar for reported cases of FcRn inhibitor use in MCr, which are summarized in Table 1 [54,55,56,57,58,59,60,61,62,63,64,65]. At present, most reports on the use of FcRn inhibitors in MCr involve EFG. Only one case has been reported using EFG-SC, and no cases using ROZ have been identified to date. Among the reported cases, seven patients (25.9%) were male, and the median age at onset of MCr was 54.75 years (range: 35–86 years), with a median disease duration of 24 months (range: 1–336 months). Thymoma-associated MG (TAMG) accounted for 14 cases (51.9%), all of whom had undergone thymectomy. Antibody status was as follows: anti-AChR antibody-positive in 77.8% (21/27), anti-MuSK antibody-positive in 18.5% (5/27), and seronegative in 3.7% (1/27). Conventional treatments for MCr were administered in 88.5% (23/26) of cases. EFG was effective in 24 of 26 cases (92.3%) in MCr, with many patients showing clear clinical improvement within or during the first treatment cycle. Additionally, the single reported case of EFG-SC also showed symptom improvement within the first cycle.

Kawama et al. reported two cases where EFG was administered following the failure of FT, but both cases were ineffective. In these cases, while total serum IgG levels decreased, anti-AChR antibody titers paradoxically increased, suggesting the possibility of an “overshoot” phenomenon induced by EFG [54]. Therefore, in addition to monitoring IgG levels, it is essential to monitor autoantibody titers during EFG treatment. Conversely, Watanabe et al. reported improvement with EFG in a patient whose symptoms worsened due to an overshoot after PLEX [55], suggesting that the possibility of overshoot does not necessarily preclude EFG use. However, clinicians should be aware of the possibility of post-treatment exacerbation and closely monitor both antibody levels and clinical status.

Zhang et al., Hong et al., and Song et al. each reported transient decreases in CD4-positive T cells and CD19-positive B cells following EFG administration, although the underlying mechanisms remain unclear [56,58,59]. Ohara et al. treated MCr without conventional FT, using only EFG in combination with IVMP. The MG-ADL score improved from 11 to 2 after one cycle of EFG, and the patient was weaned off mechanical ventilation after three doses. This report represents the first case demonstrating the acute-phase efficacy of EFG in MCr without the use of IVIg or PLEX [61]. Sorrenti et al. discussed the use of EFG in a seronegative MG (SNMG) patient with MCr, reporting a favorable response despite the drug being approved only for anti-AChR antibody-positive MG in the US and Europe [62]. Shi et al. reported four cases of MuSK-positive MG with MCr, all of which showed IgG reduction and marked clinical improvement after one cycle of EFG. However, they did not describe changes in antibody titers [63]. Shelly et al. reported a case of MCr due to paraneoplastic MG. The patient was anti-AChR antibody-positive and unresponsive to PLEX. A bladder mass was detected on CT, and the patient was diagnosed with paraneoplastic MG. EFG was administered preoperatively to stabilize symptoms. The MG-ADL score improved from 19 to 12 after one cycle of EFG, enabling surgical resection. This report suggests the potential utility of EFG for preoperative symptom control [64].

Regarding EFG-SC, Kweidor et al. reported that even the subcutaneous formulation demonstrated a rapid clinical effect in a case of MCr in TAMG with anti-AChR positivity [65]. Although this may offer a new option for patients with difficult intravenous access, further case accumulation is warranted.

The cases provide preliminary evidence that FcRn inhibition may be a promising therapeutic strategy for MCr, with rapid clinical improvement observed during the first treatment cycle, highlighting its potential for fast onset of action, although some patients did not respond. EFG-SC shows promise as a convenient subcutaneous formulation; however, reports remain very limited, and no cases for ROZ have been published to date, precluding conclusions regarding comparative efficacy among formulations.

Regarding safety and monitoring, several important observations have emerged. While theoretical concerns exist regarding infection risk due to total IgG reduction, no fatal infections have been reported in the cases examined. Some reports have described a paradoxical rise in pathogenic antibody titers (e.g., anti-AChR titers), known as the “overshoot phenomenon.” In two such cases, one involved metastatic thymoma and showed symptom improvement after ECU administration [54]. The mechanism of overshoot remains unclear but may involve antibody redistribution or transient compensatory antibody production. Clinically, sequential monitoring of both total IgG and pathogenic antibody titers is recommended. Rapid intervention using PLEX or IVIG should be performed if symptom exacerbation occurs. Some reports also suggest EFG may be effective following PLEX-induced overshoot [55], indicating that overshoot does not necessarily contraindicate EFG, but careful individualized judgment is required. Timing adjustments are critical when combining FcRn inhibitors with PLEX or IVIG, as PLEX can remove the drug itself, and IVIG may occupy FcRn, reducing EFG binding efficiency if administered beforehand, or accelerating IgG degradation if FcRn inhibitors are given first.

Immunologically, transient decreases in CD4+ T cells and CD19+ B cells have been observed after EFG administration in some reports [56,58,59]. These changes likely reflect secondary immunodynamic effects due to rapid IgG reduction rather than the direct cytotoxicity of FcRn inhibitors. The molecular mechanisms remain unclear, and the systematic evaluation of immune phenotypes and cytokine profiles is an important future research task.

Practical clinical implications are as follows: (1) FcRn inhibitors may achieve a rapid effect in MCr, but selection depends on individual clinical circumstances (e.g., prior PLEX/IVIG response, infection risk, availability of administration routes); (2) During treatment, frequent evaluation of total IgG, specific autoantibody titers, and clinical scores (e.g., MG-ADL) is recommended to prepare for overshoot or infection. Currently, most reports involve EFG; establishing the comparative effectiveness and safety of ROZ and EFG-SC requires case–control studies, registry research, or randomized trials.

The limitations of the present analysis are evident. Most data derive from case reports or series, with potential selection and reporting biases, limiting generalizability. Reported outcomes are largely short term; long-term relapse rates, immunological marker dynamics, infection risks, and survival outcomes remain insufficiently characterized. Future research should focus on multicenter prospective cohorts or registry studies to evaluate: (1) efficacy and safety; (2) time to effect, duration, and tissue distribution among agents; (3) the correlation of overshoot with predictive factors; (4) optimal combination and timing with existing therapies such as PLEX and IVIG; and (5) associations of immune phenotypes and biomarkers (complement activation markers, specific antibody subclasses, FcRn-related genetic polymorphisms) with clinical outcomes.

In conclusion, current case reports suggest that FcRn inhibitors may be a promising treatment option for MCr, but careful monitoring and preparedness for potential risks such as antibody overshoot are essential. Systematic data collection and comparative trials are urgently needed to establish optimal positioning and use algorithms for each formulation.

### 3.2. Complement Inhibitors and MCr

This section discusses the application of complement inhibitors for MCr treatments, based on reported cases. Currently, three C5-targeting complement inhibitors—ECU, RAV, and ZIL—are clinically available. RAV is a long-acting complement inhibitor that retains the antigen-binding region of ECU while modifying the Fc region to increase FcRn affinity and prolong serum half-life [49]. Administration routes and intervals differ: ECU is administered intravenously every two weeks, RAV every eight weeks, and ZIL subcutaneously on consecutive days. Both ECU and RAV are humanized monoclonal antibodies that inhibit C5 cleavage, whereas ZIL is a small synthetic peptide with a short half-life and reversible action. Due to its short half-life, ZIL is less affected by PLEX-induced drug removal, and, unlike monoclonal antibodies, it is minimally influenced by FcRn modulation, allowing co-administration with IVIG or FcRn inhibitors. In addition, dual mechanisms—C5 cleavage inhibition and blockade of C5b–C6 complex formation—effectively suppress MAC formation [50]. Consequently, ZIL can be used in cases with C5 gene variants (e.g., Arg885His) that render ECU ineffective [66].

We conducted a search of PubMed and Google Scholar for reported cases of complement inhibitors use in MCr, which are summarized in Table 2 [39,40,41,67,68,69,70,71,72,73,74,75,76]. At present, the majority of reported cases involve ECU, with 20 cases, while only 2 cases have been reported for RAV and one case for ZIL. Among the total of 23 reported patients, 11 were male (47.8%). The median age at MCr onset was 62 years (range: 24–83 years), and the median disease duration was 12 months (range: 0–288 months).

In terms of MG subtypes, TAMG accounted for eight cases (34.8%) and early-onset MG (EOMG) for four cases (17.4%). All TAMG patients and 75% (3/4) of EOMG patients had undergone thymectomy. All cases were positive for AChR-Ab, and conventional treatments for MCr were administered in every case. The use of complement inhibitors in MCr was clinically effective in 22 out of 23 patients (95.7%), with symptom improvement observed in most cases within four weeks. Notably, in cases treated with ECU, clinical improvement was often seen within one week, and, in some patients, improvement was observed as early as the day following administration. Complement-related laboratory tests were performed in six cases, all of which showed decreased complement activity [41,71,76]. In contrast, AChR antibody titers were measured in six cases; four showed no significant difference compared to placebo [71], while two showed a decrease [39,76].

Although meningococcal vaccination is mandatory prior to the use of C5 inhibitors, in the context of acute-phase treatment, there were cases where vaccination was administered concurrently with or after initiation of the complement inhibitor. Sixteen patients (69.6%) received the meningococcal vaccine prior to or simultaneously with complement inhibitor treatment. Additionally, 21 patients (91.3%) received antibiotic prophylaxis at the start of complement inhibitor administration. The antibiotics used included ceftriaxone (CTRX) in five cases, rifampicin (REP) in five cases, meropenem (MEPM) in two cases, penicillin-class agents (PCN) in two cases, sulbactam/cefoperazone (SBT/CPZ) in one case, and amoxicillin (AMPC) in one case.

The following is a detailed discussion of case reports involving ECU. Hofstadt et al. are believed to be the first to report the acute-phase use of ECU in a patient who developed MCr triggered by COVID-19 [67]. Subsequently, Usman et al. reported three cases in which ECU was administered for MCr, with all patients showing improvement in MG-ADL scores within four weeks. However, one of the cases was complicated by systemic infection and a delay in ECU administration. Although clinical improvement was observed, the extent of recovery was limited, suggesting that infection and the timing of treatment may influence therapeutic outcomes. Usman et al. also described a case in which notable improvement in respiratory function was observed one week after ECU administration [68].

Song et al. reported the clinical course of four patients treated with ECU for MCr. Notably, by 12 weeks after ECU initiation, all four cases showed reductions in CH50 and soluble C5b-9 (sC5b-9) levels. However, CH50 was not completely suppressed in all cases: in two of the four, the levels remained above the detection limit (10 U/mL). There were no significant changes in anti-AChR antibody titers before and after treatment, and no statistically significant differences were found compared with placebo. In addition, no notable changes were observed in T-cell or B-cell subsets, supporting the notion that ECU acts selectively at the terminal end of the complement cascade. One of the four cases (Case 4) was complicated by pneumonia and heart failure, and the patient exhibited a poor response to ECU. Although extubation was achieved at week 12, the patient died from heart failure at week 16 [71].

Erra et al. reported their clinical experience with ECU in 12 patients with either MCr or impending MCr. In 10 of these cases, a significant improvement in MG-ADL scores was observed within one to two weeks. These improvements were already statistically significant at weeks 1 and 2 and remained stable beyond weeks 3 and 4. The two cases that did not show improvement were both complicated by infections [74].

Takanobu et al. presented a case report and literature review on the use of ECU in patients with refractory MG who were dependent on mechanical ventilation. Among the eight cases in which successful weaning from ventilation was achieved, the median time to weaning was reported to be seven days. In contrast, weaning was difficult in two cases complicated by pneumonia, and, in another case, it required 27 days. In a large German cohort study of 250 patients with ventilator-dependent MCr treated with conventional immunotherapy, the mean duration of mechanical ventilation was 19.4 days, and 45% of patients required support for more than 15 days. These findings highlight the potential rapid therapeutic effect of ECU [39].

Next, we review case reports involving the use of RAV. Konen et al. reported the first case of RAV administration in a patient with MCr who was refractory to multiple standard therapies, including IVIg, immunoadsorption, and rituximab. Administration of RAV during the acute exacerbation phase led to significant improvements in neuromuscular symptoms—including dysphagia, respiratory function, and limb muscle strength—within one week. These improvements persisted throughout a 19-week follow-up period. Further enhancement in MG-ADL scores and quality of life was noted after a second dose of RAV [40].

Uchi et al. reported the first case in which RAV was used during MCr to achieve successful weaning from mechanical ventilation. In this case, extubation was performed on day 20 following RAV administration. The authors also examined CH50 levels as a marker of complement activity, noting that a decrease in CH50 was not observed in the early phase of treatment. CH50 levels only fell below the detection limit after the third dose. This finding aligns with the current understanding that CH50 fluctuates considerably during RAV therapy and may not serve as a reliable marker of C5 inhibition. Consequently, CH50 may not be a suitable objective indicator for evaluating the early efficacy of RAV [76].

In terms of comparing the time to extubation between RAV and ECU, the limited number of reported cases makes it difficult to draw definitive conclusions. Accumulation of further cases is warranted to clarify this issue.

Finally, we review a case report involving ZIL. Ito et al. described a patient with MCr triggered by aspiration, who showed poor short-term response to IVIg and IVMP. The patient was successfully weaned from mechanical ventilation in the acute phase following the addition of ZIL, a complement C5 inhibitor. Notable improvement in thoracic movement was observed after ZIL initiation, and extubation was achieved within four days. CH50 levels were also reported as an indicator of complement activity: although the level decreased from over 60 U/mL before treatment to 18.6 U/mL, it did not fall below the lower limit of normal. This suggests that clinical efficacy can be achieved even without CH50 levels reaching undetectable levels [41].

The cases provide preliminary evidence that C5 inhibition is a promising therapeutic strategy for MCr, with improvement observed within four weeks; sometimes as early as one week or even the day after administration [72]. However, some cases show a limited response. Rapid clinical improvement in MG-ADL and QMG scores aligns with the pathophysiological rationale that complement mediates the final common pathway of NMJ destruction. Reports on RAV and ZIL are very limited, precluding conclusions regarding inter-drug clinical differences.

Safety and monitoring considerations include: (1) post-treatment changes in anti-AChR antibody titers, which often show limited decline [39,76], indicating that C5 inhibition blocks complement-mediated damage rather than antibody production. Thus, antibody titers may not reliably indicate clinical efficacy; (2) CH50 (overall functional assessment of the complement system in serum) changes, which decreased in some cases [41,71,76] but remained near the detection limit in others [41,71], suggesting the limitations of CH50 as a sole pharmacodynamic marker. Combined assessment with sC5b-9 may improve evaluation [71]. Risk of meningococcal infection: vaccination is recommended whenever feasible. Some reports describe vaccination being administered either concurrently with, or shortly after, MCr. Prophylactic antibiotics were often co-administered. In cases where infection occurred or dosing was delayed, the clinical response was sometimes insufficient, with pneumonia or upper respiratory tract infections being the associated complications [68,74]. Delays related to the COVID-19 pandemic also led to suboptimal responses [68]. These findings underscore the importance of infection assessment, management, and appropriate dosing schedules in determining clinical outcomes.

Practical clinical implications: (1) Complement inhibition is a useful and practical option for MCr of anti-AChR antibody-positive MG, particularly with rapid response reported with ECU. (2) Treatment evaluation should combine clinical indicators with complement function markers, as efficacy does not always correlate with antibody titers. (3) Structural and pharmacokinetic differences among agents (antibody vs. peptide, half-life, FcRn dependence) influence dosing convenience, interactions with co-therapies, and efficacy in cases with specific genetic variants, guiding individualized drug selection. Timing adjustments with PLEX or IVIG are critical, as PLEX can remove both antibodies and therapeutic agents, while IVIG may affect FcRn-dependent drug retention. (4) Insufficient improvement has been reported with coexisting pneumonia or delayed administration, emphasizing timely infection management. Comparative effectiveness and safety studies among RAV, ZIL, and ECU require case–control, registry, or randomized trials.

The limitations are evident. Most data are derived from case reports or series, with selection and reporting biases limiting generalizability. Short-term evaluation predominates; long-term relapse rates, infection risk, survival, and cost-effectiveness remain insufficiently assessed. Future research should include multicenter prospective cohorts or registry studies to examine: (1) efficacy and safety; (2) comparison of time to effect, duration, and tissue distribution; (3) correlation of complement function markers (CH50, sC5b-9, etc.) with clinical outcomes; (4) biomarker-guided drug selection incorporating genetic variants (e.g., C5 mutations); and (5) optimization of combination and timing with PLEX or IVIG.

In conclusion, C5 inhibitors may be a useful option for anti-AChR antibody-positive MCr, but vaccination against meningococcal infection (when feasible), infection screening, prophylactic antibiotics, and frequent monitoring of clinical status and complement function remain essential. Systematic data collection and comparative trials are urgently needed to define the positioning of each agent and establish optimal use algorithms informed by appropriate biomarkers.

## 4. Treatment Selection in MCr

This review comprehensively summarizes the experience of using novel molecularly targeted therapies—namely, FcRn inhibitors and complement inhibitors—in MCr. Reports on their use remain limited and, although some treatment guidance has been suggested, generalizability has not yet been established. At present, there is insufficient evidence to guide the choice between these agents, and selection must be based on a comprehensive assessment of individual patient factors, including disease status, severity, comorbidities, treatment history, and drug accessibility.

**Figure 2:**
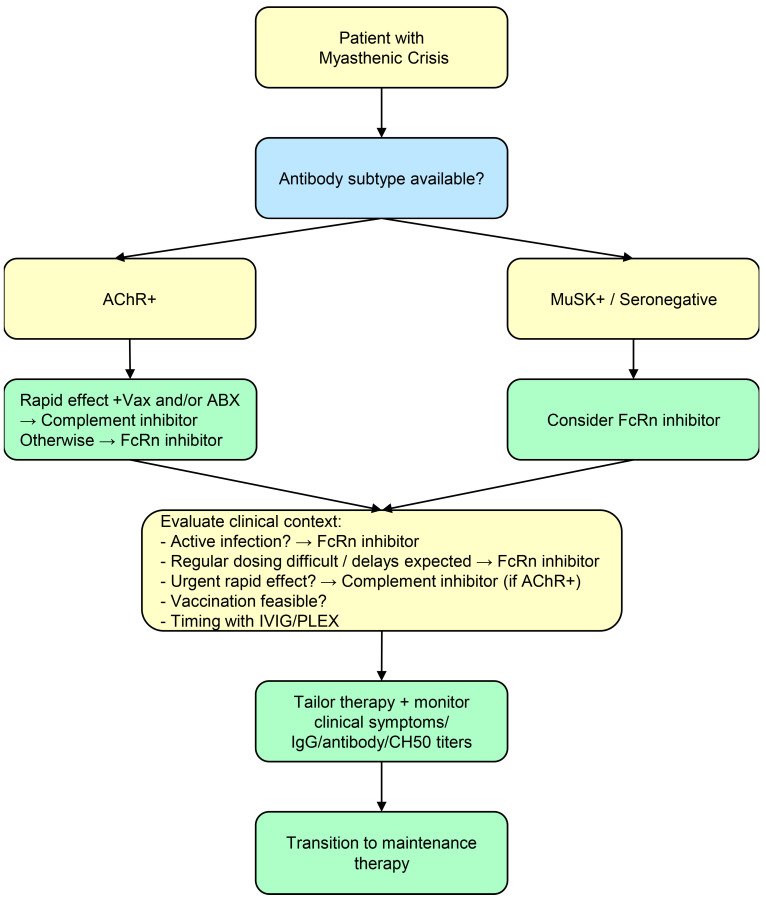
Chart showing clinical implications. AChR+ patients: complement inhibitors (rapid effect) with vaccination/antibiotic prophylaxis should be used. MuSK+ or seronegative: FcRn inhibitors. Medical professionals should consider infection, dosing feasibility, urgent symptom relief, vaccination, and IVIG/PLEX timing. Symptoms should be monitored, as well as IgG, antibody titers, and CH50, and then a transition to maintenance therapy should be undergone.

The clinical implications are as follows (Figure 2): Complement inhibitors are limited to anti-AChR antibody-positive MG and are preferably used in patients expected to achieve rapid symptom improvement. In contrast, patients with concurrent pneumonia or upper respiratory tract infections, or those in whom delays in drug administration are anticipated, may not achieve sufficient benefit and the use of these therapies should be approached with caution. In indicated cases, vaccination and prophylactic antibiotics should be considered. FcRn inhibitors can be used regardless of antibody subtype and offer flexibility for patients at high risk of infection or with diverse disease subtypes. However, the potential for post-administration antibody overshoot should be considered, and longitudinal monitoring of not only IgG levels but also specific antibody titers is recommended.

Furthermore, after improvement from MCr, patients transition to maintenance therapy. By adopting a backward-planned treatment selection strategy that considers the use of molecularly targeted therapies in the maintenance phase from MCr treatment stage, a “seamless treatment strategy” enabling smooth transition to maintenance therapy can be achieved. In designing such therapeutic strategies, multiple factors should be comprehensively considered, including the administration route and interval, pregnancy status, relevant genetic variants, preventive measures for meningococcal infection risk, and the need for concomitant IVIG or PLEX.

## 5. Conclusions

The use of molecularly targeted therapies in MCr represents a promising new option that may complement the limitations of conventional treatments. However, the current evidence is limited to case reports and small-scale studies, and robust high-level data are lacking. Future research should incorporate stratification by antibody subtype (e.g., AChR, MuSK, or LRP4), clearly defined inclusion criteria for MCr or pre-MCr states, and the longitudinal tracking of biomarkers such as anti-AChR antibody titers, CH50, and IgG subclasses. Evaluations of seamless treatment strategies that consider the transition from MCr to maintenance therapy are also essential. Ultimately, prospective randomized controlled trials and multicenter real-world registry analyses that reflect these elements will be critical for clarifying optimal treatment selection and timing, thereby advancing personalized therapy in the context of MCr.

## Figures and Tables

**Table 1:** Summary of case reports on FcRn inhibitors in MCr.

	Author	No.	Age (y)	M/F	Dur (m)	Subtype	Ab	Th-x y/n	MCr Tx	MG-ADL [QMG]				Post-MTT Params		MTT Eff.	PreMed Before MTT
										MCr Tx		MTT		IgG	Ab	Imp /Wors	Vax/ ABX
										Pre	Post	Pre	Post				
EFG	Kawama, 2023 [54]	1/2	52	M	168	TAMG	AchR	y	IVIG IVMP PLEX	NA	NA	[2]	[10]	↓	↑	Wors	NA/ NA
		2/2	53	F	120	TAMG	AchR	y	IVIG	NA	NA	[4]	[11]	↓	↑	Wors	NA/ NA
	Watanabe, 2024 [55]	1/1	54	F	5	TAMG	AchR	y	IVIG IVMP PLEX	17	20	20	21 (1 cy) 1 (3 cy)	↓	↓	Imp	NA/ NA
	Zhang, 2024 [56]	1/1	35	F	3	TAMG	AchR	y	IVIG IVMP	18 [26]	20 [27]	19 [27]	3 [13] (1 cy)	↓	↓	Imp	NA/ NA
	Omar, 2024 [57]	1/1	57	M	NA	NA	AchR	NA	PLEX	21 [29]	20 [26]	20 [26]	3 [11] (1 cy)	NA	NA	Imp	NA/ NA
	Hong, 2024 [58]	1/1	58	F	96	LOMG	AchR	n	IVIG IVMP	NA	17 [24]	17 [24]	5 [9] (3rd time in 1 cy)	↓	↓	Imp	NA/ NA
		1/2	60	F	108	TAMG	AchR	y	IVIG	NA	14 [23]	14 [23]	7 [14] (2nd time in 1 cy)	↓	↓	Imp	NA /NA
		1/3	66	F	84	TAMG	AchR	y	—	NA	14 [21]	14 [21]	6 [6] (1 cy)	↓	↓	Imp	NA /NA
	Song, 2024 [59]	10	55.5	M4 F6	3.9 ± 8.1 (y)	TAMG6 gMG3 Musk1	AchR9 Musk1	y6 n4	NA	NA	NA	15.6 ± 4.4	3.4 ± 2.2 (1 cy)	↓	↓	Imp	NA/ NA
	Morita, 2024 [60]	1/1	38	M	NA	TAMG	AchR	y	IVIG IVMP PLEX	10	14	14	2 (1 cy) 0 (7 cy)	↓	↓	Imp	NA/ NA
	Ohara, 2024 [61]	1/1	70	F	1	LOMG	AchR	n	—	NA	NA	11	2 (1 cy) 1 (4 cy)	↓	↓	Imp	NA/ NA
	Sorrenti, 2024 [62]	1/1	56	F	336	SNMG	—	n	IVIG PLEX	NA	11	11	3 (1 cy) 0 (5 cy)	↓	↓	Imp	NA/ NA
	Shi, 2024 [63]	1/1	63	F	24	Musk-MG	Musk	NA	IVIG PLEX RTX	NA	NA	5	0 (1 cy)	↓	NA	Imp	NA/ NA
		1/2	42	F	24	Musk-MG	Musk	NA	PLEXRTX	NA	NA	13	4 (1 cy)	↓	NA	Imp	NA/ NA
		1/3	51	F	3	Musk-MG	Musk	NA	IVIG IVMP RTX	NA	NA	18	7 (1 cy)	↓	NA	Imp	NA/ NA
		1/4	43	F	24	Musk-MG	Musk	NA	—	NA	NA	14	3 (1 cy)	↓	NA	Imp	NA/ NA
	Shelly, 2025 [64]	1/1	86	F	4	LOMG	AchR	NA	PLEX	23	21	19	12 (1 cy) 3 (4 cy)	NA	NA	Imp	NA/ NA
EFG-SC	Kweidor, 2024 [65]	1/1	54	F	180	TAMG	AchR	y	—	NA	NA	10 [18]	3 [3] (1 cy)	↓	↓	Imp	NA/ NA

Ab = Antibody; ABX = Antibiotic prophylaxis; AchR = Acetylcholine receptor; cycle = cy; Dur = Disease Duration; Eff = Effect; EFG = Efgartigimod; EOMG = Early-onset MG; gMG = Generalized MG; Imp = Improve; IVIg = Intravenous Immunoglobulin; IVMP = Intravenous methylprednisolone pulse therapy; LOMG = Late-onset MG; MCr = Myasthenic crisis; MG = Myasthenia Gravis; MG-ADL = Myasthenia Gravis-Activities of Daily Living; MTT = Molecularly targeted therapy; Musk = Muscle-specific kinase; n = no; NA = Not Applicable; No. = Number; Params = Parameters; PLEX = Plasma exchange; Pre-Med = Pre-Medication; QMG = Quantitative Myasthenia Gravis; RTX = Rituximab; SC = Subcutaneous injection; SNMG = Seronegative MG; TAMG = Thymoma-associated MG; Thx = Thymectomy; Tx = Treatment; Vax = Vaccination; Wors = Worsen; y = yes.

**Table 2:** Summary of case reports on complement inhibitors in MCr.

	Author	No.	Age (y)	M/F	Dur (m)	Subtype	Ab	Th-x y/n	MCr Tx	MG-ADL [QMG]				Post-MTT Params		MTT Eff.	PreMed Before MTT
										MCr Tx		MTT		CH- 50	Ab	Imp/Wors	Vax/ABX
										Pre	Post	Pre	Post				
ECU	Hofstadt, 2021 [67]	1/1	62	M	9	LOMG	AchR	NA	IVIG PLEX RTX	NA	NA	7	2 (2 wk) 0 (11 wk)	NA	NA	Imp	NA/y CTRX
	Usman, 2021 [68]	1/3	24	F	132	EOMG	AchR	y	IVIG RTX	NA	13	12	4 (4 wk) 0 (5 wk)	NA	NA	Imp	Men/y
		2/3	77	M	1	LOMG	AchR	n	IVIG PLEX	NA	NA	20	3 (5 wk) 0 (24 wk)	NA	NA	Imp	Men/y
		3/3	56	M	12	LOMG	AchR	n	IVIG PLEX	NA	NA	24	20 (3 wk) 14 (40 wk)	NA	NA	Imp	Men/y
	Strano, 2022 [69]	1/1	48	M	7	TAMG	AchR	y	IVIG PLEX	NA	NA	23	17 (4 wk)	NA	NA	Imp	Men/ CTRX
	Vinciguerra, 2023 [70]	1/1	74	M	24	LOMG	AchR	n	IVIG	NA	NA	NA	NA	NA	NA	Imp	n/RFP
	Song, 2024 [71]	1/1	71	M	2	LOMG	AchR	n	IVIG PLEX	NA	15 [15]	15 [15]	0 [5] (4 wk) 0 [3] (12 wk)	↓	no sig diff. vs. pbo	Imp	n/ SBT/CPZ
		1/2	34	F	120	EOMG	AchR	y	PLEX	NA	19 [26]	19 [26]	3 [6] (4 wk) 0 [3] (12 wk)	↓		Imp	n/n
		1/3	37	M	4	TAMG	AchR	y	IVIG PLEX	NA	18 [24]	18 [24]	10 [22] (4 wk) 1 [9] (12 wk)	↓		Imp	n/CTRX
		1/4	64	F	5	TAMG	AchR	y	IVIG IVMP	NA	21 [24]	21 [24]	17 [22] (4 wk) 8 [15] (12 wk)	↓		Imp	n/MEPM
	Messina, 2025 [72]	1/1	72	M	0	LOMG	AchR	n	IVIG PLEX	16	20	20	16 (1 d) 5 (2 wk)	NA	NA	Imp	Men/y
	Durmus, 2025 [73]	1/1	73	F	0	LOMG	AchR	n	IVIG	24	22	21	6 (4 wk)	NA	NA	Imp	Men/ MEPM
		1/2	61	F	72	TAMG	AchR	y	IVIG	24	24	13	0 (4 wk)	NA	NA	Imp	Men/PCN
		1/3	37	F	2	TAMG	AchR	y	IVIG	24	24	17	1 (4 wk)	NA	NA	Imp	Men/PCN
	Erra, 2025 [74]	1/1	32	F	108	TAMG	AchR	y	IVIG	24 [23]	24 [23]	24 [23]	4 [8] (4 wk)	NA	NA	Imp	Men/RFP
		1/2	72	M	144	TAMG	AchR	y	IVIG PLEX	24 [33]	24 [33]	24 [33]	24 [33] (4 wk)	NA	NA	Wors	Men/RFP
		1/3	74	M	24	LOMG	AchR	n	IVIG	24 [39]	24 [39]	24 [39]	4 [8] (4 wk)	NA	NA	Imp	Men/RFP
		1/4	78	M	288	LOMG	AchR	n	IVIG	24 [22]	24 [22]	24 [22]	1 [13] (4 wk)	NA	NA	Imp	Men/RFP
	Takenobu, 2025 [39]	1/1	38	F	3	EOMG	AchR	y	IVIG IVMP	24	24	24	18 (2 d) 4 (4 wk)	NA	↓	Imp	Men/NA
	Rossini, 2025 [75]	1/1	32	F	132	TAMG	AchR	y	PLEX	6 [12]	23 [29]	23 [29]	20 [24] (1 wk) 2 [12] (3 wk)	NA	NA	Imp	Men/ CTRX
RAV	Konen, 2024 [40]	1/1	34	F	8	EOMG	AchR	n	IVIG PLEX	11 [18]	[25]	[23]	4 (7 wk) 2 (15 wk) [6] (2 wk)	NA	NA	Imp	n/AMPC
	Uchi, 2024 [76]	1/1	71	F	24	LOMG	AchR	n	IVMP	[14]	NA	NA	[9] (3 wk)	↓	↓	Imp	Men/n
ZIL	Ito, 2025 [41]	1/1	83	F	17	LOMG	AchR	n	IVIG IVMP	13	23	23	18 (10 d) 9 (23 d)	↓	NA	Imp	Men/ CTRX

ABX = Antibiotic prophylaxis; AchR = Acetylcholine receptor; AMPC = amoxicillin; CTRX = ceftriaxone; d = day; diff. = difference; ECU = Eculizumab; Eff. = Effect; EOMG = Early-onset MG; Imp = Improve; IVIg = Intravenous Immunoglobulin; IVMP = Intravenous methylprednisolone pulse therapy; LOMG = Late-onset MG; Men = Meningococcal; MEPM = meropenem; MCr = Myasthenic crisis; MG = Myasthenia Gravis; MG-ADL = Myasthenia Gravis-Activities of Daily Living; MTT = Molecularly targeted therapy; n = no; NA = Not Applicable; No. = Number; pbo = placebo; PCN = penicillin-class agents; PLEX = Plasma exchange; QMG = Quantitative Myasthenia Gravis; REP = rifampicin; RTX = Rituximab; SBT/CPZ = sulbactam/cefoperazone; sig = significant; TAMG = Thymoma-associated Myasthenia Gravis; Tx = Treatment; Vax = Vaccination; wk = weeks; Wors = Worsen; y = yes.

## Data Availability

No new data were created or analyzed in this study. Data sharing is not applicable to this article.

## Abbreviations

AChR = acetylcholine receptor, ECU = eculizumab, EFG = efgartigimod, EFG-SC = subcutaneous efgartigimod, FcRn = neonatal Fc receptor, IVIG = intravenous immunoglobulin, IVMP = intravenous methylprednisolone, LRP4 = low-density lipoprotein receptor-related protein 4, MAC = membrane attack complex, MCr = myasthenic crisis, MG = myasthenia gravis, MG-ADL = Myasthenia Gravis—Activities of Daily Living, MuSK = muscle-specific kinase, NMJ = neuromuscular junction, PLEX = plasma exchange, QMG = Quantitative Myasthenia Gravis, RAV = ravulizumab, ROZ = rozanolixizumab, VNTRs = variable number tandem repeats, ZIL = zilucoplan.
